# Functionalization of Zeolite NaP1 for Simultaneous Acid Red 18 and Cu(II) Removal

**DOI:** 10.3390/ma14247817

**Published:** 2021-12-17

**Authors:** Tomasz Bień, Dorota Kołodyńska, Wojciech Franus

**Affiliations:** 1 Faculty of Geology, Geophysics and Environmental Protection, AGH University of Science and Technology, Al. Adama Mickiewicza 30, 30-059 Kraków, Poland; tomasz.bien@op.pl; 2 Biko-Serwis sp. z o.o. sp.k., ul. Zakładowa 13, 26-052 Nowiny, Poland; 3 Department of Inorganic Chemistry, Faculty of Chemistry, Institute of Chemical Sciences, Maria Curie-Skłodowska University, M. Curie Skłodowska Sq. 2, 20-031 Lublin, Poland; d.kolodynska@poczta.umcs.lublin.pl; 4 Department of Construction Materials Engineering and Geoengineering, Faculty of Civil Engineering and Architecture, Lublin University of Technology, Nadbystrzycka 40, 20-618 Lublin, Poland

**Keywords:** fly ash, zeolite, chitosan, dyes, intermolecular interactions

## Abstract

The efficiency of azo dye Acid Red 18 (AR18) and Cu(II) ions simultaneous removal from an aqueous solution on NaP1CS and NaP1H was investigated, taking into account the effect of the phase contact time, pH, initial concentration, temperature, and interfering ions presence. Zeolite denoted as NaP1CS was modified by chitosan (CS) and zeolite denoted as NaP1H was modified by hexadecyltrimethylammonium bromide (HDTMA). In order to characterize sorption properties of NaP1CS, the obtained sorbent was characterized using Fourier transform infrared spectroscopy (FTIR) and nitrogen adsorption/desorption (ASAP). The kinetic parameters were determined by means of the pseudo first order (PFO), pseudo second order (PSO), and intraparticle diffusion (IPD) kinetic models. To present the adsorption data, three different isotherm models (Langmuir, Freundlich and Dubinin-Radushkevich) were used. The desorption process was also examined. It was found that for sorbent NaP1CS the pseudo second order (PSO) kinetic model and the Langmuir isotherm fitted best the experimental data. Moreover, it was noted that the acidic pH is appropriate to achieve the best sorption properties of NaP1CS for Cu(II) and NaP1H for AR18 and Cu(II). The thermodynamic parameters indicate an endothermic process. The most effective solution for the desorption process was found to be 1 M HCl. The results indicate that simultaneous removal of dye AR18 and Cu(II) on modified zeolite NaP1CS or NaP1H is possible and proceeds with a very good efficiency. The obtained zeolites could effectively adsorb AR18 an Cu(II) simultaneously, but their adsorption abilities were rather different.

## 1. Introduction

Nowadays, synthetic dyes are much more often used than natural ones [1]. Large amounts of colored wastewaters are produced by such industries as the textile, paper, and plastic industries [2,3]. Dyes can cause allergies and are often characterized by toxic and carcinogenic properties which impose threat for human health [4,5]. The presence of dyes in surface waters can give negative effects (such as confined access to the light, which results in the inhibition of photosynthesis, thus disrupting proper functioning of aquatic ecosystems) [4,6]. Furthermore, unfortunately synthetic dyes are not degradable and stable, so removal of dyes from wastewaters is an urgent task [7]. Not only the presence of dyes in wastewaters poses a threat to aquatic ecosystems and human health, but also heavy metal ions have negative impact [7,8]. Since industrial wastewaters contain both dyes and heavy metal ions, there is a need to examine the systems containing both pollutants simultaneously.

The largest group of synthetic dyes with one or more azo group (–N=N–) contains about half of annual worldwide production of colorants [6,9]. They are used even in the paper, cosmetic, food, and drug industries. 

Acid Red 18, denoted as (AR18) and commonly known as Ponceau 4R, Cochineal Red A, or New Coccine is one of the such synthetic azo dyes [10,11]. It is mainly used in the food manufacturing industry as a coloring agent E124 [12]. It is also applicable in the drug, cosmetics and pharmaceutical industries [13]. In many cases it occurs simultaneously with metal ions. There are many known methods of wastewater treatment from both heavy metals and dyes e.g. adsorption, ion exchange, coagulation and flocculation, ozonation, reverse osmosis, membrane filtration, activated sludge, chemical oxidation, electrodialysis, and even monopolar electro-coagulation process [14,15,16]. Also, advanced oxidation processes (e.g., the Fenton process with formation of ferrous ions nanoscale zero-valent iron (NZVI)) can be used for this aim. According to the study of Nazaria et al. [9], the dye removal efficiency (%S) was about 34% and 98% for H_2_O_2_ (200 mM) and NZVI/H_2_O_2_ (NZVI and H_2_O_2_ concentrations were 2 g/L and 200 mM, respectively), at a contact time of 80 min and pH 3. When the pH value increased to 9, then %S decreased to 12% and 29% for H_2_O_2_ and NZVI/ H_2_O_2_, respectively.

However, most of these methods are not sufficiently effective due to the chemical inertness of most dyes. As shown by some studies in the case of exceeding a daily dose AR18 can adversely influence on human health resulting in neurobehavioral effects, intake reproductive toxicity, mutagenic action, and potential carcinogenicity [17]. Dyes also cause eutrophication and interference in ecology and chemical changes in water streams [6]. The World Health Organization (WHO) and Food and Agriculture Organization (FAO) regulate daily consumption of AR18 in an amount less than 4.0 mg/kg.

In the case of Cu(II) ions concentrations in drinking water, they vary widely as a result of variations in water characteristics, such as pH, hardness, and its availability in the distribution system. According to the literature data, it was found that Cu(II) levels in drinking water can range from ≤0.005 mg/L to >30 mg/ L [17]. Therefore, for simultaneous dyes and metal ions removal, adsorption is considered as one of the best techniques due to low cost, effectiveness and easy handling [8,15,18]. For this purpose, carbon based materials (activated carbons, biochars, and polymeric based materials) are used [5,19]. Searching for proper material with good adsorption properties, scientists turned to zeolites.

Zeolites are crystalline aluminosilicates with a skeleton made up of tetrahedral SiO_4_ and AlO_4_ [7,20,21]. They are characterized by excellent ion exchange, adsorption and catalytic properties owing to a number of channels and pores in their structures [22]. Zeolites have structural negative charge and high affinity for transition metal cations. The chemical composition of the zeolite unit cell is expressed by the formula: M_n_O·Al_2_O_3_·xSiO_2_·yH_2_O, where M is the cation of an alkali metal, *n* is the valence charge, ad *x* and *y* are the integers [23]. The excess negative charge is compensated by ions of sodium, calcium, magnesium and potassium. Synthetic zeolites have significantly better adsorption properties than natural ones [23].Therefore, they are the object of greater interest. In most cases these materials can be modified by incorporation of various functional groups such as hydroxyl, carboxylic, amine, thiol, phosphate, crown ether, etc. These sorbents are chiefly used for heavy metal ions removal [24,25,26]. Functional groups grafted to the zeolite matrix improve their selectivity not only towards heavy metal ions but also dyes. To introduce these functionalities, chitosan and HDTMA were used [27].

Chitosan (CS) is a cationic polysaccharide obtained by alkaline N-deacetylation of chitin. However, contrary to chitin, the presence of a large number of amine groups on the chitosan chain increases its adsorption capacity [28]. Protonated amino groups can interact electrostatically with anionic dyes in acidic media. Furthermore, acetamide and hydroxyl groups present in the chitosan can serve as active sites [27]. Thus modification of zeolites by chitosan can improve the ability to remove heavy metals and dyes [29]. Furthermore, chitosan modified glass beads [30], activated clays [31], silica [32], and polymers are well described [1]. Carbon nanotubes (CNT) were used as a matrix for preparation of CS/CNT composites for dyes removal by Wang et al. [33].

This paper presents zeolite (NaP1) obtained from fly ash by the hydrothermal method and then modified by chitosan (NaP1CS) and hexadecyltrimethylammonium bromide (HDTMA) (NaP1H) as perfect adsorbent for simultaneous removal of AR18 and Cu(II) [34]. It is well-known that the adsorption of cations and especially of anions on the surfaces of zeolites is very limited. However, the anion exchange capacity (AEC) of zeolites can be improved by chemical modification of their surface properties using selected organic compounds such as HDTMA. Therefore, the influence of experimental conditions such as pH, phase contact time, initial concentration, temperature, and interfering ions presence on effectiveness of sorption on chitosan and HDTMA modified zeolite NaP1CS and NaP1H were studied. Moreover, the interactions between AR18 and Cu(II) as well as CS or HDTMA and zeolite were characterized.

## 2. Materials and Methods

### Materials

For synthesis of zeolite there was used fly ash (result of combustion of bituminous coal) from the power generating plant “Kozienice” (Manufacturer, Kozienice, Poland). Zeolite NaP1 was produced based on hydrothermal synthesis of fly ash with sodium hydroxide at atmospheric pressure [35]. Synthesis was performed on a pilot-scale installation for 24 h at 353 K as described in [36]. Chitosan (CS) used in the studies was obtained from Sigma Aldrich (chitosan flakes with the deacetylation degree > 75%). Acid Red 18 (AR18) dye was also purchased from Sigma Aldrich. The general characteristics of the dye used in the research are presented in Table 1. Hexadecyltrimethylammonium bromide (HDTMA) (Merck) was used at amounts equivalent to 1.0 and 2.0 of the NaP1 cation exchange capacity (CEC) according to the procedure described in [34].

The first stage of adsorbent NaP1 modification was to dissolve CS flakes in 1% solution of glycolic acid. It lasted for 24 h using a magnetic stirrer at 1000 rpm at room temperature. In the next stage zeolite NaP1 was added to CS solution, with the ratio of 8:1 and then the mixture was continuously blended using a magnetic stirrer at 1000 rpm for 6 h. To precipitate the resulting product the 1 M NaOH solution was used. Subsequently it was filtered and washed with distilled water to neutral pH, dried and then ground to obtain NaP1CS. In the case of HDTMA modification, zeolite NaP1 was dispersed in about 300 mL of deionized water and the desired amount of HDTMA was slowly added with the ratio of 1:1 and then the mixture was continuously blended using a magnetic stirrer at 1000 rpm for 2 h.

To characterize the sorbents the Fourier transform infrared spectroscopy (FTIR) method is used. FTIR spectra were obtained using a Cary 630 FTIR Spectrometer (Agilent Technologies). The Fourier transform infrared spectra of the samples were measured at 650–4000 cm^−1^. Another analysis was applied to determine such parameters as: specific surface area, micropore surface, micropore volume, total pore volume and average pore diameter. The measurements of N_2_ adsorption/desorption isotherms at 77 K were conducted by ASAP 2420 (Micromeritics Inc., Norcross, GA, USA). To determine the surface morphologies of the sorbents SEM images were taken using the Quanta 3D FEG (FEI) electron microscope.

Stock solution of Cu(II) with the concentration 1000 mg/L was prepared by dissolving proper amounts of CuCl_2_·2H_2_O (obtained from Avantor Performance Materials Poland S.A.) in distilled water. All of the chemicals used were of analytical grade and used without further purification. The stock solutions of Cu(II) and AR18 were prepared by directly dissolving them in distilled water.

To determine the optimal pH for the sorption of the dye the first tests were performed for the system containing only the dye. The studies were carried out under acidic (pH = 3, 4), almost neutral (pH = 6) and alkaline conditions (pH = 9). For the system containing the AR18 and Cu(II) at concentration (50 mg/L AR18 and 50 mg/L Cu(II)) the effect of pH was studied after adjustement of the initial pH of the solution using 1 M NaOH and 1 M HCl. To determine the effect of pH, 20 mL of the appropriate solution were added to 0.1 g of NaP1, NaP1CS or NaP1H and shaken for 120 min. at room temperature with the amplitude equal 7 and 180 rpm.

**Figure 1:**
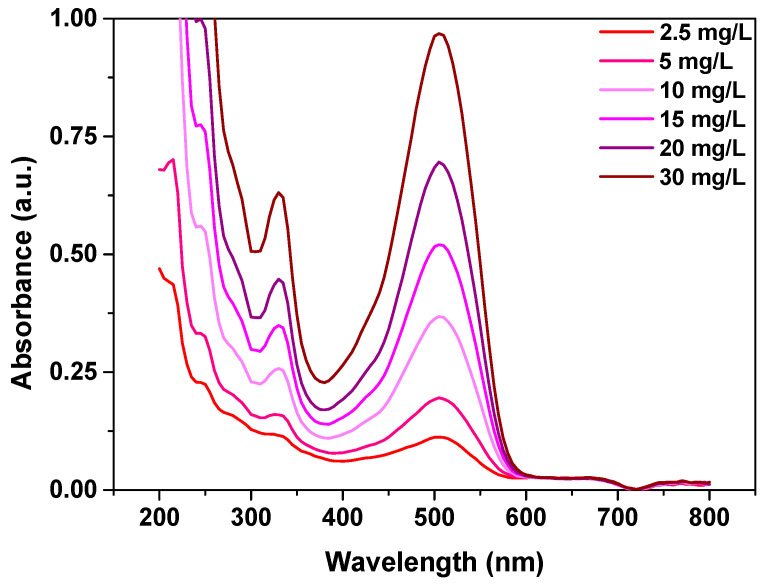
Spectra of AR18 at different concentrations.

In each test, the samples after shaking were filtered and the concentration of Cu(II) was determined by means of the AAS technique using the spectrometer SpectrAA FS-240 (Manufacturer, Varian, Bungarra, Australia). Concentration of the dye solution was measured using UV-Vis spectrophotometer, from Agilent Technologies (Santa Clara, CA, USA), Cary 60 at an optimal wavelength of 506 nm. Figure 1 presents the spectra of dye AR18, at different concentrations.

Kinetic experiments for AR18 and Cu(II) on NaP1, NaP1CS and NaP1H sorption were carried out by mixing 0.1 g of sorbent with 20 mL two-component solution at concentrations: (50 mg/L AR18 and 50 mg/L Cu(II) and 100 mg/L AR18 and 100 mg/L Cu(II)). Solutions with sorbents were mixed for the period of time 1–240 min with the shaking amplitude 7 and 180 rpm at 293 K. To calculate the kinetic parameters the pseudo first order, pseudo second order and intraparticle diffusion kinetic models were used. The pseudo first order equation is generally expressed as follows [36]:

(1) $$\frac{dq_{t}}{dt}=k_{1}\left(q_{e}-q_{t}\right)$$

where *q_e_* and *q_t_* are the adsorption capacities at equilibrium and at time *t*, respectively (mg/g), *k*_1_ is the rate constant of pseudo first order model (1/min).

After integration and applying the boundary conditions, *t* = 0 to *t* = *t* and *q_t_* = 0 to *q_t_* = *q_t_*, this equation is as follows:

(2) $$q_{t}=q_{e}\left(1-e^{-k_{1}t}\right)$$

The pseudo second order adsorption kinetic rate equation is expressed as follows:

(3) $$\frac{dq_{t}}{dt}=k_{2}{\left(q_{e}-q_{t}\right)}^{2}$$

where *k*_2_ is the rate constant of the pseudo second order model (g/mg min) and *q_e_* is the adsorption capacity calculated by the pseudo second order model (mg/g).

Integrating Equation (3) and applying the boundary conditions, that is, *t* = 0 to *t* = *t* and *q_t_* = 0 to *q_t_* = *q_t_*, gives [27]:

(4) $$q_{t}=\frac{k_{2}q_{e}^{2}t}{1+k_{2}q_{e}t}$$

Furthermore, the diffusion model was also considered so as to determine the rate-limiting step during the overall adsorption process. One well-known type of diffusion equations used to model the adsorption process is given by Weber-Morris [37]

(5) $$q_{t}=k_{i}t^{1/2}+C$$

where *q_t_* is the adsorption capacity (mg/g) at time *t*, *t* is the contact time (min), both *k_i_* (mg/g min^0.5^) and *C* (mg/g) are the Weber−Morris diffusion constants.

In this paper, three different isotherm models were investigated for representing the adsorption data including the Langmuir, Freundlich and Dubinin-Radunshkevich models. The initial AR18 and Cu(II) concentrations were varied from 25–400 mg/L. 20 mL of the appropriate solution was added to 0.1 g of the NaP1CS and NaP1H and mixed at the shaking time 120 min, the shaking amplitude was 7 and 180 rpm at 293 K. The most useful isotherm is the Langmuir isotherm model which indicates monolayer and a homogeneous surface with no interactions between the adsorbate molecules [38]. The Langmuir equation is as follows:

(6) $$q_{e}=\frac{q_{m}K_{L}c_{e}}{1+K_{L}c_{e}}$$

where *q_e_* is the adsorption capacity (mg/g) at equilibrium, *c_e_* is the adsorbate equilibrium concentration in solution (mg/L), *q_m_* is the monolayer adsorption capacity of the sorbent (mg/g) and *K_L_* is the Langmuir constant (L/mg) related with the sorption free energy [39]. From the linear plot of *c_e_/q_e_* vs. c_e_ both *q_m_* and *K_L_* can be determined.

Another model is the Freundlich model, which is an empirical isotherm for sorption on heterogeneous surfaces and multilayer sorption. It is presented in the equation below:

(7) $$q_{e}=K_{F}c_{e}^{1/n}$$

where *K_F_* (mg/g) is a constant relating the adsorption capacity and 1/*n* is an empirical parameter relating the adsorption intensity, which varies with the heterogeneity of the material [39]. In turn, the Dubinin–Radushkevich (D–R) isotherm model assumes multilayer sorption of ions in the most energetically favourable sites of sorbent [40]. The Dubinin-Radunshkevich isotherm is expressed by Equation (8) [41]:

(8) $$q_{e}=q_{s}e^{\left(-Bs^{2}\right)}$$

which *q_s_* and *B* constants are obtained from the intercept and slope of the experimental plot of *lnq_e_* vs. *ε*^2^. *ε* is the Polanyi potential and can be calculated from:

(9) $$\varepsilon =RTln\left(1+\frac{1}{c_{e}}\right)$$

where *R* is the gas constant (8.314 J/mol K), *T* is the temperature (K), *c_e_* is the equilibrium concentration of the adsorbate (mg/L), *q_s_* is the sorption monolayer capacity, the parameter *B* can be used to calculate the mean free energy of sorption from Equation (10):

(10) $$E=2B^{-1/2}$$

Temperature effect on adsorption capacity of AR18 and Cu(II) on NaP1CS and NaP1H was studied at 293, 313, and 333 K using 400 mg/L of the initial two-component solution. To explain the effect of temperature on the adsorption process there were used such thermodynamic parameters as the Gibb’s energy change (Δ*G*°), enthalpy (Δ*H*°), and entropy (Δ*S*°). Free energy (Δ*G*°) was calculated using the following equation [41]:

(11) $$\Delta G{}^{\circ}=-RTln\left(K_{c}\right)$$

where *R* is the gas constant (8.314 J/mol K), *T* is the temperature (K), and *K_C_* is the equilibrium constant equal to *q_e_/c_e_*. Δ*H*° and Δ*S*° were calculated from the slope and intercept of van’t Hoff plots of ln*K*c vs. 1/*T.*

To determine the interfering ions effect the multi-component solution at the concentrations 50 mg/L AR18 and 50 mg/L Cu(II) with the addition of Cl^−^, NO_3_^−^, SO_4_^2−^ anions at the concentrations 1000 mg/L were prepared. 20 mL of the appropriate solution was added to 0.1 g of NaP1CS or NaP1H and mixed together at the shaking time 120 min., the amplitude 7, 180 rpm and the temperature 293 K.

Desorption process was examined using 96% C_2_H_5_OH, 99.8% CH_3_OH, 1 M HCl, 1 M CH_3_COOH, 1 M NaOH, and 1 M NaCl solution. 20 mL of solution for desorption was poured into the Erlenmeyer flask containing 0.1 g of NaP1Cs and NaP1H after the adsorption equilibrium of AR18 and Cu(II). The process was carried out for 120 min. at room temperature with the shaking amplitude 7 and 180 rpm. The desorption percentage D(%) of AR18 and Cu(II) was defined as [42]:

(12) $$D\left(\%\right)=\frac{c_{e\left(des\right)}}{c_{e\left(ads\right)}}$$

where *c_e(des)_* is the concentration of AR18 and Cu(II) desorbed from NaP1CS or NaP1H (mg/L) and *c_e(ads)_* is the concentration of AR18 and Cu(II) adsorbed on NaP1CS or NaP1H (mg/L).

## 3. Results

### 3.1. Chemical Characterization of the Materials

**Figure 2:**
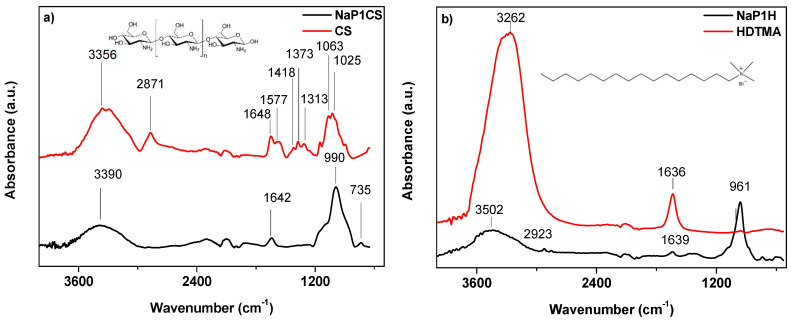
FTIR spectra of (**a**) CS and NaP1CS as well as (**b**) HDTMA and NaP1H.

The Fourier transform infrared spectra of the samples were measured at 650–4000 cm^−1^. Figure 2a presents the spectra of chitosan (CS) and NaP1CS. Figure 2b presents the spectra of hexadecyltrimethylammonium bromite (HDTMA) and NaP1H.

Major adsorption bands for chitosan are situated at 3356 cm^−1^ (–OH and –NH_2_ stretching vibrations), 2871 cm^−1^ (–CH stretching vibrations), 1648 and 1577 cm^−1^ (–NH_2_ bending vibrations), 1418 and 1313 cm^−1^ (C–O stretching vibrations), 1373 cm^−1^ (C–N stretching vibrations), 1063 and 1025 cm^−1^ (C–O skeletal vibrations) [42]. Appearance of the bands of NaP1CS spectra at 735 and 990 cm^−1^ indicates the presence of Al–O–Si and Si–O bonds stretching, respectively, derived from zeolite. Moreover, after modification of zeolite with CS, it can be observed in the spectra that the intensities of the hydroxyl peaks and amide peaks decrease and are found at 3390 cm^−1^ and 1642 cm^−1^ respectively. In the case of HDTMA modification the following bands were found: 3502 cm^−1^ and 2923 cm^−1^ (asymmetric and symetric vibrations of surfactant ‘head’ suggesting that it possesses the quaternary ammonium groups –N(CH_3_)_3_ as well as at 1639 cm^−1^ connected with CH_3_–N band. It shoud be noted that it also contains 16 carbons and thus being relatively longer than other quaternary ammonium salts, is usually used for modifying zeolites. As for a role of HDTMA, it is not bound to the silicate surfaces.

**Figure 3:**
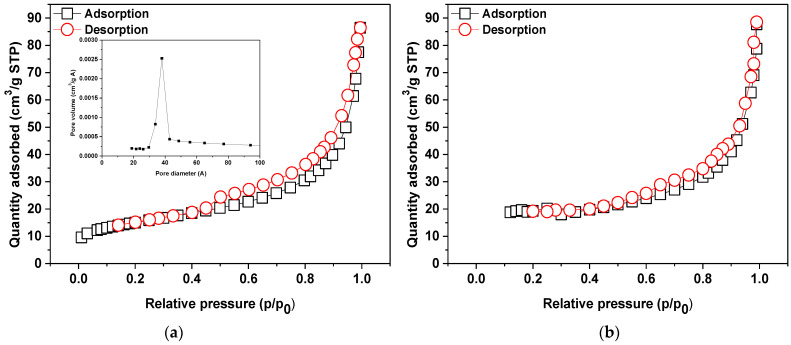
N_2_ adsorption–desorption isotherms at 77 K and BJH desorption pore size distribution of (**a**) NaP1CS and (**b**) NAP1H.

Another analysis was applied to determine such parameters as: specific surface area, micropore surface, micropore volume, total pore volume and average pore diameter. Table 2 presents values of surface area, pore size and pore volume. According to the IUPAC classification the N_2_ adsorption/desorption isotherms of NaP1CS belong to IV type isotherm and H_2_/H_3_ hysteresis loop [43]. The mesopore width reported in the literature is 20 to 500 Å so the pore distribution curves are typical of mesopores. Analogous results were obtained for NaP1H. Examplary results for NaP1CS were presented in Figure 3.

The analysis of textural parameters revealed that both modifications reduce S_BET_ and porosity of the materials. Consequently, all examined parameters confirm this trend apart from D_av_. The surface of the NaP1 zeolite after modification was covered with an organic phase (HDTMA or CS) causing pore blockage. The total pore volume of NaP1 was equal to 0.28 cm^3^/g, while after the modification process, the total pore volume achieved the value 0.13 and 0.11 cm^3^/g for NaP1CS and NaP1H, respectively. However, the average pore diameter increased for NaP1CS to 111.7 Å and decreased for NaP1H to 65.4 Å, in comparison to NaP1 (109.1 Å). These data confirm that long chains of HDTMA are more effective in reducing the textural performance of NaP1 than CS. 

**Figure 4:**
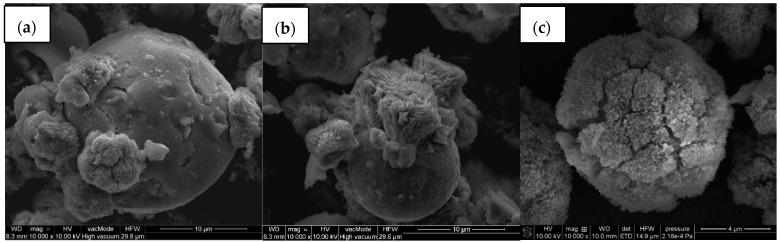
SEM photomicrographs of the (**a**) NaP1, (**b**) NaP1CS and (**c**) NaP1H (magnification 10,000×).

The SEM images Figure 4 show that the particles of zeolite NaP1 assumed a spherical shape of different sizes and other irregular forms. After modification the surface was covered by CS or HDTMA.

In the case of XRD pattern for NaP1 and modified NaP1, NaP1H was prepared according to the procedure described in [34]. Muir et al. mentioned that the analysis of the XRD pattern did not show any changes and no additional peaks were observed after the modification of NaP1 by HDTMA. This indicates that the ion exchange process was responsible for the adsorption of surfactant onto the zeolite’s surface and thus the structural perturbations did not take place.

### 3.2. pH Effect

**Figure 5:**
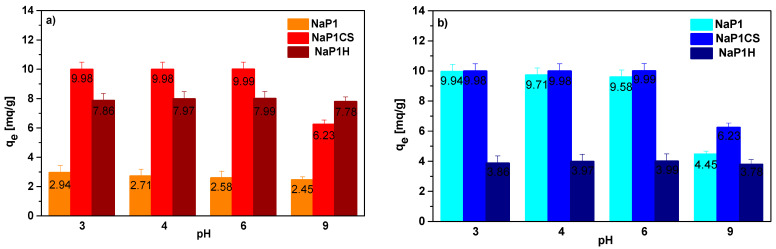
Influence of pH on adsorption of (**a**) AR18 and (**b**) Cu(II) on NaP1, NaP1CS and NaP1H (m = 0.1 g, t = 120 min, A = 7, 180 rpm, pH = 3–9).

The effect of pH on the adsorption was investigated at the AR18 and Cu(II) concentration 50 mg/L for 240 min. The effect of the pH on AR18 and Cu(II) adsorption on NaP1CS and NaP1H was studied over the pH range from 3 to 9. Examining the sorption of the dye, it was observed that the percentage of adsorption (%S) in an acidic medium at pH 3 was equal to 41% for NaP1 and 97% for NaP1CS and 89% for NaP1H while in the alkaline medium at pH 9 the adsorption percentage (%S) decreased (data not presented). The obtained values relate to the *q_e_* were presented in Figure 5.

For zeolite NaP1 after sorption of AR18 *q_e_* values dropped insignificantly from 2.94 mg/g to 2.45 mg/g, whereas for chitosan modified zeolite NaP1CS were higher but also dropped from 9.98 mg/g to 6.23 mg/g. In the case of HDTMA modified zeolite NaP1H they were lower compared to NaP1CS and dropped from 7.86 mg/g to 7.78 mg/g. The adsorption capacity for Cu(II) on NaP1CS and NaP1H do not change with pH value increasing. In the range of pH value from 3 to 6, these values were equal almost 10 mg/g and decreased at pH 9 to 6.23 mg/g for NaP1CS (Figure 5b). At a pH above 9, blue flocs appeared with increasing the pH value of AR18 and Cu(II) solution. This is probably due to the hydrolysis process and Cu(II) hydroxide precipitation. The results indicate that acidic pH is effective in achieving maximum dye removal. This is due to the fact that the pH value influences on CS and HDTMA surface charge. With the reduction of pH, the number of protonated amino functional groups of CS on the NaP1 surface increases that can interact electrostatically with an anionic dye AR18. In alkaline solution, the amine groups of CS are deprotonated, so electrostatic interaction between the NaP1CS and AR18 was reduced and resulted in lower removal efficiency. The mechanism of AR18 adsorption on chitosan modified NaP1 can be illustrated by the following steps:

- Protonation (−NH_3_^+^) amino groups of chitosan (−NH_2_) under acidic conditions (Equation (13))
  NaP1CS + H^+^ ⇄ NaP1CS H^+^– (13)
- Simultaneously dissociation of dye molecule (D–SO_3_^−^), as shown in Equation (14):
  D–SO_3_Na ⇄ D–SO_3_^−^ + Na^+^ (14)
- The electrostatic interactions between NaP1CS H^+^ and D–SO_3_^−^ (Equation (15))
  NaP1CS H^+^ + D–SO_3_^−^ ⇄ NaP1CS H^+^ + O_3_S–D (15)

It was estimated that the adsorption of Cu(II) can proceed on zeolite modified CS as a result of electrostatic interactions in the acidic media (ion exchange) or metal chelation.

In the case of HDTMA, it not only makes NaP1 surface more hydrophobic but also neutralizes the negative charges. It is well-known that HDTMA bilayer on NaP1 surface affect the AR18 adsorption. The values of HDTMA^+^ parametres: diameter 0.4 nm, length 2.3 nm and polar head diameter 0.694 nm compared to NaP1 channels suggest slight changes in external cation exchange capacity. Thus, the possible mechanism of AR18 dye adsorption onto NaP1H occurs only on the outer surface. Examining the two-component solution, it was found that pH change from 3 to 6 has a slight effect on the efficiency of AR18 and Cu(II) sorption as shown in Figure 5. As it can be seen from Figure 5, the maximum sorption of AR18 by NaP1H takes place in acidic condition (pH = 3). This effect of pH can be explained with regards to the interaction between AR18 and HDTMA in terms of surface charge. The AR18 is an acidic dye and its sulfonate moiety contains negative sulfonic groups (–SO_3_^−^). In acidic condition, a layer of HDTMA on the surface of zeolite increases the positive charges on the external surface of zeolite. Therefore, the strong electrostatic attraction between the positively charged sorption site and oppositely charged groups of the AR18 molecules leads to high adsorption capacity of AR18. The noticeable decrease in the AR18 sorption capacity can be noticed by increasing pH. The appropriate pH value was equal to 6.0. It was found that CS modification NaP1 is characterized by better properties than HDTMA modified and therefore further investigations using HDTMA were not caried out. Summarising, modification of the NaP1 zeolite with chitosan (NaP1CS) increases Cu(II) and AR18 sorption, while modification of the NaP1 zeolite with HDTMA (NaP1H) increases AR18 sorption and decreases Cu(II) sorption.

### 3.3. Effect of Initial Concentration

**Figure 6:**
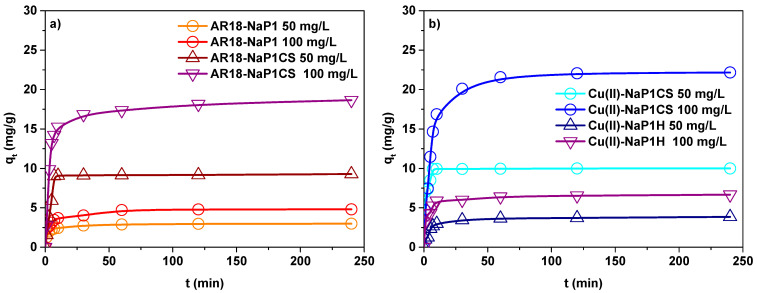
Concentration effect of (**a**) AR18 and (**b**) Cu(II) adsorption on NaP1CS (m = 0.1 g, t = 120 min, A = 7, 180 rpm, pH = 6).

Since the preliminary results indicated that for NaP1CS the maximum adsorption capacities at simulataneous AR18 and Cu(II) removal were achieved at pH 6, in the next step the influence of initial concentration was tested at this value. Influence of initial concentration of AR18 and Cu(II) on adsorption was examined in the two-component solution at two different concentrations 50 and 100 mg/L. As shown in Figure 6 the adsorption capacity increased with the increasing concentration of AR18 and Cu(II).

### 3.4. Kinetic Effect

The obtained data were modelled using the pseudo first order, pseudo second order and intraparticle diffusion kinetic models. Table 3 presents the parameters for three kinetic models of adsorption of AR18 and Cu(II) on NaP1 and NaP1CS. This data showed that experimentally calculated values of *q_e_* for the tested concentrations were identical with the theoretical calculated ones in the case of the pseudo second order kinetic model [44]. Furthermore, the values of determination coefficients R^2^ were also the highest for the pseudo second order model. This indicates that the pseudo second order kinetic model fits better the adsorption process than the pseudo first order kinetic model. The same trend is observed in many papers. A big difference between the equilibrium constant k_2_ and k_1_ indicates that the NaP1CS surface was heterogeneous [45]. 

According to the Weber-Morris equation, the adsorption process can be controlled in three different stages. The first stage is the rapid external surface adsorption, while the second step is related to the intraparticle diffusion. The third one is the final equilibrium stage, where intraparticle diffusion starts to slow down [22,46]. This can be due to adsorbate concentration in aqueous solutions and a smaller number of available adsorption sites. The values of k_i_ and C presented in Table 3 were calculated from the second section of the plot. The values of k_i_ and C for the adsorption of AR18 and Cu(II) on NaP1 and NaP1CS increased with the increasing initial AR18 and Cu(II) concentration. However, the intraparticle diffusion model did not fit the adsorption process due to the non-linearity of the plots [32].

### 3.5. Adsorption Isotherms

**Figure 7:**
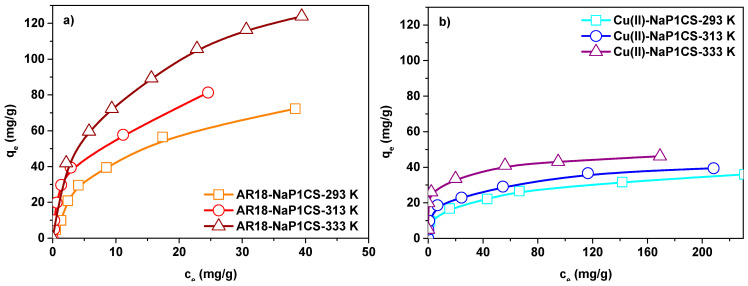
Effect of temperature on the adsorption isotherms of (**a**) AR18 and (**b**) Cu(II) adsorption on NaP1CS (m = 0.1 g, t = 120 min, co = 10–400 mg/L, A = 7, 180 rpm, pH = 6).

In the description of experimetal data concerning the adsorption of AR18 and Cu(II) on NaP1CS there were investigated three different isotherm models: Langmuir, Freundlich, and Dubinin-Radushkevich. In Table 4 isotherm parameters were collected based on the isotherms presented in Figure 7.

The equilibrium adsorption capacities for AR18 and Cu(II) increased from 1.12 mg/g and 4.07 mg/g to 46.22 mg/g and 123.63 mg/g when AR18 and Cu(II) concentration increased from 50 to 400 mg/L, respectively. Based on the data, R^2^ values for the Langmuir model are higher than for the other model which indicates that the Langmuir model fits best the experimental data. These results show that the surface of the adsorbent is monolayer and homogeneous. Based on the Langmuir isotherms, the values of *q_m_* for both AR18 and Cu(II) ions increase with the increasing temperature which indicates that higher temperatures facilitate the adsorption process. The maximum values capacity of NaP1CS at pH 6.0, two-component solution at 400 mg/L of AR18(VI) and Cu(II) is 123.62 mg/g as for AR18 and 46.22 mg/g as for Cu(II). It is far more than that of adsorbents reported in [10,37,47]. The K_L_ constant of the Langmuir parameters demonstrated the binding affinity between NaP1CS and AR18 and Cu(II). The K_L_ values of AR18 range from 0.189 to 0.390 and for Cu(II) from 0.059 to 0. 1690. The K_L_ values suggested that NaP1CS possess stronger adsorption of AR18 than for Cu(II). Although the determination coefficients R^2^ for the Langmuir isotherm concerning the adsorption of AR18 on NaP1CS decrease with the increasing temperature. This points out that at higher temperature the Langmuir isotherm tends toward the Freundlich adsorption model. The nature of adsorption can be determined by R_L_ value. The value of R_L_ indicates whether the type of isotherm is unfavorable adsorption (R_L_ > 1), favorable adsorption (0 < R_L_ < 1), irreversible adsorption (RL = 0), or linear adsorption (R_L_ = 1) [48]. Also the n values from the Freundlich model are between 1 and 10 which confirms the favourable character of adsorption [43]. 

Determined by the Dubinin – –Radushkevich isotherm, the value of mean free energy of adsorption E predicts the mechanism of sorption. Since the values of E are between 9.659 and 16.789 kJ/mol, the ion exchange mechanism takes place (Table 4) [43].

### 3.6. Effect of Temperature

The easily accessible sorption sites and high surface area of the adsorbent contributed to the rapid adsorption. The AR18 and Cu(II) adsorption capacity for NaP1CS increased with an increase in temperature from 293 to 333 K, indicating better adsorption at higher temperature and an endothermic adsorption process. Similar results were also found concerning adsorption of AR18 on the chitosan/carbon nanotube [33]. In contrast, the AR18 adsorption capacity for NaP1 decreases with an increase in temperature from 293 to 333 K, indicating better adsorption at lower temperature and an exothermic adsorption process. Table 5 presents the thermodynamic parameters for the adsorption of AR18 and Cu(II) on NaP1CS.

The positive Δ*H*° values of AR18 adsorption on NaP1CS and Cu(II) adsorption on NaP1CS indicate that the process is endothermic [48]. The magnitude of the Δ*H*° value for physical adsorption is in the range of 2.1–20.9 kJ/mol while for chemical adsorption 80–200 kJ/mol. The Δ*H*° values of Cu(II) adsorption on NaP1CS indicate physical adsorption processes. The Δ*H*° value of AR18 adsorption on NaP1CS was in the range of neither physical adsorptions nor chemical adsorptions, which can indicate it involves the electrostatic interactions [41]. The negative and went down values of Δ*G*° at all temperatures indicate the spontaneous nature of the adsorption process of AR18 and Cu(II) ions on NaP1CS. The positive values of ΔS° mean decrease of the randomness at the solid-solution interface during the adsorption [49].

### 3.7. Interfering Ions Effect

**Figure 8:**
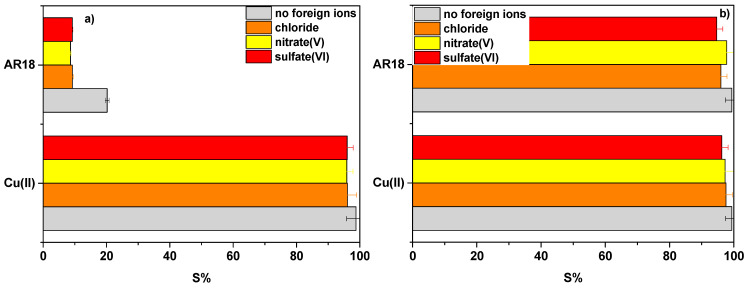
Effect of Cl^−^, NO_3_^−^, SO_4_^2−^ ions on the sorption percentage S(%) of AR18 and Cu(II) ions on (**a**) NaP1 and (**b**) NaP1CS, (m = 0.1 g, t = 120 min, A = 7, 180 rpm).

The effects of interfering ions were studied in the presence of chloride, nitrate(V) and sulfate(VI) ions (Figure 8). Their presence hardly affected sorption of AR18 and Cu(II) ions on NaP1CS in opossite to AR18 on NaP1. The greatest influence of the interfering ions was observed in the case of AR18 sorption onto NaP1.

Sorption on NaP1 was mostly affected by the presence of nitrate(V) ions decreasing from 20.2% to 8.6% for the sorption of Acid Red 18 and from 98.8% to 95.9% for the sorption of Cu(II). It can be concluded that even in the presence of chloride, nitrate(V) and sulfate(VI) sorption on NaP1CS can proceed without interference with still very high efficiency as shown in Figure 8. Chitosan modified NaP1 zeolite (NaP1CS) has a high potential in the removal of metal ions and dyes, since it has both amine (–NH_2_) and hydroxyl (–OH) group that can serve as active sites. With the reduction of pH, the number of protonated amino functional groups of CS on the NaP1CS surface increases and they can interact electrostatically with an anionic dye AR18. In alkaline solution, the amine groups of CS are deprotonated, so electrostatic interaction between the NaP1CS and AR18 was reduced and resulted in lower removal efficiency. In the case of Cu(II), it was estimated (Fig. 5) that the adsorption of Cu(II) can proceed on zeolite NaP1 and modified NaP1CS as a result of electrostatic interactions (ion exchange) or metal chelation.

### 3.8. Desorption Process

**Figure 9:**
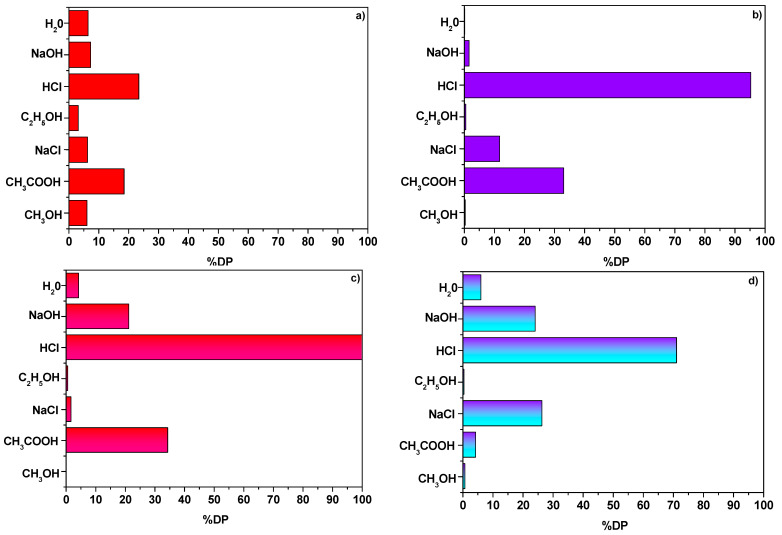
(**a**) Percentage of desorption DP(%) (**a**) AR18 and (**b**) Cu(II) from NaP1 a well as (**c**) AR18 and (**d**) Cu(II) from NaP1CS (m = 0.1 g, t = 120 min, A = 7, 180 rpm).

Recovery of heavy metal ions and molecules of dye was made possible as a result of the desorption process. Figure 9 presents the percentage of AR18 and Cu(II) ions desorption DP(%) from NaP1 and NaP1CS. Of all solution used for this purpose the most effective one proved to be 1 M HCl. DP(%) of NaP1 using 1 M HCl were 23.4% and 100% for AR18 and Cu(II) ions, respectively. In contrast, much higher values were obtained with respect to the desorption of NaP1CS and they were 71.3% and 95.2% for AR18 and Cu(II) ions, respectively. The main mechanism of AR18 adsorption on NaP1CS was ion exchange which is generally characterized by high efficiency regeneration [37].

## 4. Conclusions

In this paper, modified NaP1 zeolite by CS and HDTMA denoted as NaP1CS and NAP1H were investigated to verify the sorption properties compared with those of zeolite NaP1. Acid Red 18 was chosen as a model dye (AR18) and Cu(II) ions as the example of heavy metal ions for removal. Modification of NaP1 by chitosan (CS) and hexadecyltrimethylammonium bromide (HDTMA) improved the sorption properties towards AR18 and the sorption capacity increased almost three times compare to NaP1. However, better results were obtained for CS. Modification of the NaP1 zeolite with chitosan (NaP1CS) increases Cu(II) and AR18 sorption, while modification of the NaP1 zeolite with HDTMA (NaP1H) increases AR18 sorption and decreases Cu(II) sorption. The results showed that the increasing concentration of AR18 and Cu(II) promotes the increase of sorption capacity. The kinetic data were consistent with the pseudo second order kinetic model which is confirmed by the value of correlation coefficient R^2^. The Langmuir isotherms were the best fitted with the equilibrium data so this fact indicates monolayer and homogeneous surface of the adsorbent. The mean free energy of adsorption E indicates that the ion exchange mechanism takes place. The thermodynamic parameters indicate that temperature increase has a favourable effect on the simultaneous sorption of AR18 and Cu(II) ions. As follows from the results acidic pH is effective in achieving maximum dye removal. The presence of interfering ions did not cause decrease in the effectiveness of the adsorption process which facilitates removal of dyes and heavy metal ions. The 1 M HCl solution proved to be the most effective for the desorption process as the efficiency amounting 71.3% and 95.2% for AR18 and Cu(II) ions, respectively.

## Figures and Tables

**Table 1:** General characteristics of C.I. AR18.

Parameter	Value
Molecular formula	C_20_H_11_N_2_Na_3_O_10_S_3_
Molecular weight, g/mol	604.5
COD of 1 g AR18, mg/L	597 ± 17
λ_max_, nm	507
Chemical structure	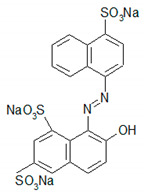

**Table 2:** Surface characteristics of NaP1CS.

Parameter	NaP1	NaP1CS	NaP1H
S_BET_ (m^2^/g)	94	53	29
V_mic_^a^ (cm^3^/g)	0.005	0.004	0.003
S_mic_^a^ (m^2^/g)	16.0	10.7	8.34
V_tot_^b^ (cm^3^/g)	0.28	0.13	0.11
D_av_^c^ (Å)	109.1	111.7	65.4

S_BET_ is the specific surface area; V_mic_ is the micropore volume; S_mic_ is the surface of micropores; V_tot_ is the total pore volume; D_av_ is the average pore diameter; ^a^ Calculated from t-plot; ^b^ Determined at p/po = 0.99; ^c^ Barrett, Joyner and Halenda (BJH) model.

**Table 3:** Kinetic parameters for the adsorption of AR18 and Cu(II) on NaP1 and NaP1CS.

Adsorbent	Adsorbate	C_0_ (mg/L)	q_e_, exp, (mg/g)	Kinetic Model	Kinetic Parameters		
				pseudo first order	q_max_ (mg/g)	k_1_ (h^−^^1^)	R^2^
NaP1	AR18	50 100	2.18 5.30		3.96 2.73	0.025 0.023	0.75 0.96
	Cu(II)	50 100	9.91 19.60		18.85 2.23	0.005 0.035	0.80 0.94
NaP1CS	AR18	50 100	9.94 19.83		1.78 1.84	0.012 0.022	0.87 0.73
	Cu(II)	50 100	9.98 19.30		99.43 4.05	0.023 0.043	0.58 0.98
				pseudo second order	q_max_ (mg/g)	k_2_ (g/mg h)	R^2^
NaP1	AR18	50 100	2.18 5.30		2.18 5.39	0.630 0.029	1.00 0.99
	Cu(II)	50 100	9.91 19.60		9.91 19.61	1.371 0.406	1.00 1.00
NaP1CS	AR18	50 100	9.94 19.83		9.93 19.84	0.173 0.029	0.99 1.00
	Cu(II)	50 100	9.98 19.30		9.98 19.39	16.632 0.045	1.00 0.99
				intra-particle diffusion	k_i_ (mg/g·min^0.5^)	C (mg/g)	R^2^
NaP1	AR18	50 100	2.18 5.30		0.243 0.276	1.35 2.45	0.97 0.90
	Cu(II)	50 100	9.91 19.60		0.003 0.265	9.85 18.71	0.81 0.99
NaP1CS	AR18	50 100	9.94 19.83		0.111 0.425	9.13 18.27	0.90 0.99
	Cu(II)	50 100	9.98 19.30		0.028 1.011	9.91 14.11	0.99 0.97

**Table 4:** Isotherm parameters for AR18 and Cu(II) adsorption on NaP1CS.

Adsorbate	Isotherm	T (K)	Parameters			
AR18	Langmuir		q_m_ (mg/g)	K_L_ (L/mg)	R^2^	R_L_
		293	74.61	0.390	0.98	0.093
Cu(II)			36.04	0.051	0.98	0.440
AR18		313	81.33	0.355	0.98	0.101
Cu(II)			39.47	0.090	0.97	0.307
AR18		333	123.63	0.189	0.87	0.175
Cu(II)			46.22	0.169	0.98	0.192
AR18	Freundlich		K_f_ (mg/g)	n	R^2^	
		293	16.43	2.48	0.53	
Cu(II)			7.67	3.57	0.98	
AR18		313	18.41	1.96	0.88	
Cu(II)			9.48	3.67	0.96	
AR18		333	18.38	1.38	0.97	
Cu(II)			15.16	4.59	0.80	
AR18	Dubinin-Radushkevich		q_s_ (mg/g)	B (mol^2^/kJ^2^)	R^2^	E (kJ/mol)
		293	0.003	0.0033	0.52	12.344
Cu(II)			1200.354	0.0025	0.98	14.031
AR18		313	0.007	0.0041	0.91	11.024
Cu(II)			1056.565	0.0024	0.97	14.469
AR18		333	0.017	0.0054	0.97	9.659
Cu(II)			1008.093	0.0018	0.80	16.789

**Table 5:** Thermodynamic parameters for Acid Red 18 and Cu(II) adsorption on NaP1 and NaP1CS.

**Adsorbent**	**Adsorbate**	**Δ*H*°** **(** **kJ/mol)**	**ΔS^°^** **(J/mol K)**	**Δ*G*° (kJ/mol)**		
				293 K	313 K	333 K
NaP1CS	AR18	29.84	106.3	−18.37	−20.88	−24.99
	Cu(II)	12.49	26.4	−2.15	−13.57	−5.53

## Data Availability

The data presented in this study are available on request from the corresponding author.
